# Impact of Marine Aquaculture on the Microbiome Associated with Nearby Holobionts: The Case of *Patella caerulea* Living in Proximity of Sea Bream Aquaculture Cages

**DOI:** 10.3390/microorganisms9020455

**Published:** 2021-02-22

**Authors:** Giorgia Palladino, Simone Rampelli, Daniel Scicchitano, Margherita Musella, Grazia Marina Quero, Fiorella Prada, Arianna Mancuso, Anne Mette Seyfarth, Silvia Turroni, Marco Candela, Elena Biagi

**Affiliations:** 1 Unit of Microbiome Science and Biotechnology, Department of Pharmacy and Biotechnology (FaBiT), Alma Mater Studiorum—University of Bologna, 40126 Bologna, Italy; giorgia.palladino2@unibo.it (G.P.); simone.rampelli@unibo.it (S.R.); daniel.scicchitano2@unibo.it (D.S.); margherita.musella2@unibo.it (M.M.); silvia.turroni@unibo.it (S.T.); marco.candela@unibo.it (M.C.); 2 Fano Marine Center, The Inter-Institute Center for Research on Marine Biodiversity, Resources and Biotechnologies, Viale Adriatico 1/N, 61032 Fano, Italy; fiorella.prada2@unibo.it (F.P.); arianna.mancuso2@unibo.it (A.M.); 3 Institute for Biological Resources and Marine Biotechnology, National Research Council (IRBIM-CNR), 60125 Ancona, Italy; grazia.quero@irbim.cnr.it; 4 Marine Science Group, Department of Biological, Geological and Environmental Sciences, University of Bologna, Via Selmi 3, 40126 Bologna, Italy; 5 Deptartment of Global Surveillance, National Food Institute, Technical University of Denmark, 2800 Kgs. Lyngby, Denmark; amse@food.dtu.dk

**Keywords:** gastropoda, patella, marine holobionts, microbiome, aquaculture, 16S rRNA gene sequencing

## Abstract

Aquaculture plays a major role in the coastal economy of the Mediterranean Sea. This raises the issue of the impact of fish cages on the surrounding environment. Here, we explore the impact of aquaculture on the composition of the digestive gland microbiome of a representative locally dwelling wild holobiont, the grazer gastropod *Patella caerulea*, at an aquaculture facility located in Southern Sicily, Italy. The microbiome was assessed in individuals collected on sea bream aquaculture cages and on a rocky coastal tract located about 1.2 km from the cages, as the control site. *Patella caerulea* microbiome variations were explained in the broad marine metacommunity context, assessing the water and sediment microbiome composition at both sites, and characterizing the microbiome associated with the farmed sea bream. The *P. caerulea* digestive gland microbiome at the aquaculture site was characterized by a lower diversity, the loss of microorganisms sensitive to heavy metal contamination, and by the acquisition of fish pathogens and parasites. However, we also observed possible adaptive responses of the *P. caerulea* digestive gland microbiome at the aquaculture site, including the acquisition of putative bacteria able to deal with metal and sulfide accumulation, highlighting the inherent microbiome potential to drive the host acclimation to stressful conditions.

## 1. Introduction

Fish farming is rapidly increasing in the Mediterranean Sea in order to respond to the rising demand for products for human consumption. Marine aquaculture (mariculture) is an integral part of growing coastal economy and is mainly carried out by caged open systems, with the farmed species in direct contact with the wild coastal ecosystem [1,2,3]. Current finfish farming practices influence the marine biota at different trophic levels by changing environmental conditions in the surrounding water column, which undergoes severe eutrophication, as well as by impacting the chemical features of the sediments below the cages [1,4,5]. Indeed, sediments in proximity of the cages show an increase in organic matter, due to the sedimentation of uneaten feed and fish feces, as well as an accumulation of heavy metals [6,7]. Consequences are shifts in nutrients and carbon fluxes, pH decline, and oxygen depletion in the sea floor, resulting in ammonia and hydrogen sulfide accumulation [4,8]. Such conditions modify the benthic assemblages of fauna and seagrass, affecting the whole food web [9]. It has been demonstrated that the proximity of fish farming cages affects the survival of grazers and other macro-fauna trophic groups, even further than the mere sedimentation zone [1,10].

All environments on our planet, including macro-organisms themselves (defined as holobionts), are colonized by microorganisms living in complex communities called microbiomes. All key biosphere processes, both terrestrial and aquatic, as well as many physiological aspects of animal and plant biology, deeply rely on microbiomes. Being aware of the microbiome importance as a life support system for the planet biosphere, there is now a huge concern about the impact of local and global anthropogenic factors on the planet microbiomes [11], making microbiome assessment a central point for a next-generation and more holistic evaluation of environmental health. In this scenario, the impact of aquaculture on seafloor microbiomes has recently been explored, reporting decreased bacterial biodiversity in the sediments below the cages, the overgrowth of microbial groups able to thrive in anaerobic, carbon-enriched conditions, as well as an accumulation of fecal bacteria and/or bacteria linked to the sulfur cycle [6,12,13,14,15,16,17]. The microbial community associated with the water column also appeared to be different within and outside the cage of farmed sea breams [18]. On the contrary, very little is known on the effect of the presence of farming cages on the microbiome of nearby wild organisms.

Animals and plants are not autonomous entities but need to be viewed as complex biomolecular networks composed by the host and its associated microbiome [19]. Microbiome structures result from a host-driven selection process. Nonetheless, in the case of marine holobionts that live in close, water-mediated interaction with microbiomes from the environment and the other holobionts living in their proximity, host-associated microbial communities show a strong metacommunity behavior. In other words, the microbiome of a given host is presumably linked by dispersal to all other microbiomes in the same environment, and microbiome recruitment by one host can be affected by the exudate of another, nearby dwelling holobiont [20,21,22,23]. Therefore, it is reasonable to expect that the microbiomes associated with wild marine holobionts living in proximity to the fish farms are somehow affected by the interaction with the microbiomes of farmed fish, both directly, by the transfer of fish microorganisms dispersed to the water column and/or sediments, and indirectly, by changing the surrounding environmental bacterial community. Eventually, the consequent colonization of the wild holobiont microbiomes with allochthonous microbial components would result in compositional changes with cascade impacts on the health and safety of the marine environment. In agreement with these hypotheses, it was recently reported that sponges living in proximity of fish farming sites in the Philippines harbor a microbial community enriched in genes involved in ammonia oxidation compared to sponges of the same species collected in pristine waters [24].

In order to explore how the presence of fish farming cages influences the microbiome of the surrounding wild holobionts, we selected a common grazer gastropod from the genus Patella as a representative fouling holobiont. *Patella caerulea* is a common seaweed grazing marine limpet in all Mediterranean rocky shores [25]. As a result of their wide distribution, abundance, and sedentary lifestyle, limpets of this species have been proposed as biomonitors for the local water quality in terms of heavy metal accumulation and organic pollutants [26,27]. In addition, limpets are keystone species for the coastal ecosystem because they regulate the degree of algal coverage and, consequently, succession processes in rocky intertidal communities [26,27,28].

In this study, we compared (by next-generation sequencing 16S rDNA metabarcoding) the *P. caerulea* digestive gland microbiome structure in individuals collected close to sea bream (*Sparus aurata*) aquaculture cages located in a fish farm in Southern Sicily (Italy), Mediterranean Sea, with the one from individuals collected on a rocky coastal tract located at 1.2 km from the aquaculture facility, as a control site. The gut, skin, and gill microbiomes from the farmed fishes were also assessed, together with microbial assemblages from sediments and water at the aquaculture and control sites, allowing us to explore the variation of *P. caerulea* microbiomes at the aquaculture site in a holistic metacommunity context. Both sampling sites were located in the harbor of Licata, which is an ideal location to investigate the impact of aquaculture systems on the microbiome composition of nearby wild animals due to the limited hydrodynamic circulation inside the harbor and to its shallow depth (≈10 m), resulting in a large amount of organic matter accumulating on the sea floor under the cages [14]. This comprehensive study design allowed us to dissect the interaction between the microbiomes from farmed fishes and surrounding wild holobionts at the metacommunity level, showing patterns of microbial dispersion from the former to the environment and, finally, to locally dwelling wild organisms.

## 2. Materials and Methods

### 2.1. Site Description, Samples Collection, and Environmental Data

**Figure 1:**
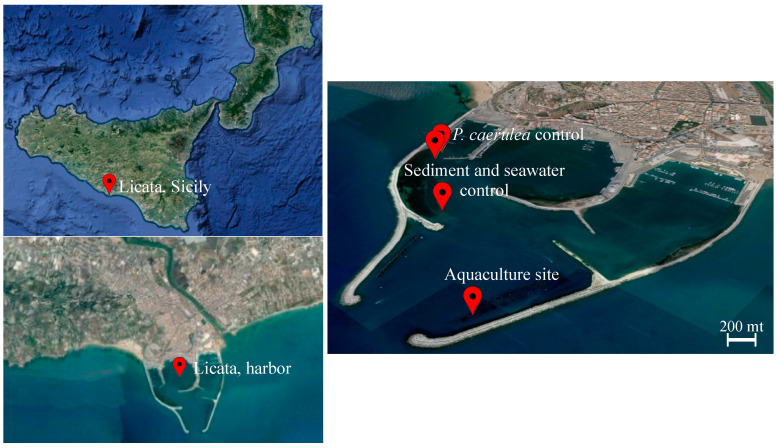
Sampling site description. Map of the marine fish farm located in the harbor of Licata (37.087713° N, 13.943773° E). The sampling sites are indicated with red balloons and labeled accordingly. The aquaculture site is located at 37.086667° N, 13.943611° E, control sediment, and water samples were collected far from the fish cages (37.089732° N, 13.937469° E and 37.091949° N, 13.933703° E), and *P. caerulea* individuals from the shallow water rocks located along the pier were collected at 37.092222° N, 13.933056° E (source: Google Earth, earth.google.com/web/; last access: 10 January 2021; map data: SIO, NOAA, U.S. Navy, NGA, GEBCO, IBCAO).

The sampling was performed on 25 September 2019, in a marine fish farm located in the harbor of Licata (Figure 1), in Southern Sicily (Mediterranean Sea, coordinates 37.087713° N, 13.943773° E). The facility is composed of 23 floating cages containing separately sea bream (*S. aurata*) and sea bass (*Dicentrarchus labrax*); further details on the sampling site are reported in Ape et al. [14]. Sea bream and sea bass have productive cycles of different length; thus, at the time of sampling, only sea bream cages contained a high number of adult fishes, representing a large biomass potentially influencing the local limpet microbiome. Indeed, in September, *S. aurata* individuals were almost at the end of their productive cycle, causing a progressive transformation of the benthic substrate into a muddy black sediment and making the sampling period ideal for the maximization of the aquaculture plant impact on the wild environment. We selected one of the sea bream cages as the sample site (37.086667° N, 13.943611° E), and we collected five *S. aurata* individuals and 12 *P. caerulea* individuals, the latter growing in adhesion to the cage plastic tubes. Surface sediment (0–1 cm) and seawater were collected under the cage as well as at two additional sites (37.089732° N, 13.937469° E and 37.091949° N, 13.933703° E, about 670 m and 1.2 km far from the aquaculture site, respectively) as controls, by coring and through a sterile plastic bottle, respectively. Limpet samples were detached using a previously sterilized knife and preserved into sterile plastic containers. We also collected 15 *P. caerulea* individuals from the shallow water rocks located along the pier (37.092222° N, 13.933056° E, 1.2 km far from the aquaculture site), as described above. From each fish, intestinal content (gut), gills, and skin were collected. In more detail, sea bream individuals (average 270 g) were euthanized by anesthesia (MS-222) following the national regulations and set on ice until processing. Within 2–5 h, gut tissues were obtained by aseptic dissection, and the intestinal content was squeezed out as described in Mente et al. [29] and stored in sterile tubes. Finally, a 2 × 2 cm of skin (left side) and the second gill branch (left side) were aseptically collected with sterile scissors, rinsed with sterile phosphate buffer, and stored in sterile tubes. All samples were kept at −20 °C until shipping in the respective labs.

Seawater temperature and pH measurements were performed with an Onset HOBO Data Logger. Salinity was measured through an optical refractometer. Water samples for determination of total alkalinity (TA) were collected at both sites using sterile 120 mL syringes. The syringe samples were immediately transferred in labeled 100 mL amber glass bottles and fixed with saturated mercuric chloride (HgCl_2_) to avoid biological alteration and stored in darkness at 4 °C prior to measurement. TA was determined by potentiometric titration (6 measurements per site) using a Metrohm 888 Titrando inter-faced with the data acquisition software TiAmo Light. The HCl (0.1 M) titrant solution was calibrated against certified reference materials distributed by A.G. Dickson (CRM, Batches #187). Seawater pH, TA, salinity, and temperature were used to calculate other carbonate chemistry parameters using the software CO2SYS with referenced dissociation constants [30,31,32].

### 2.2. Microbial DNA Extraction, 16S rRNA Gene Amplification, and Sequencing

For limpet samples, we dissected the digestive gland from each individual under a vertical laminar airflow cabinet using sterile tweezers and scalpels, obtaining a weight range from 0.037 to 0.460 g for all the glands, depending on each limpet size. Total microbial DNA extraction was performed on limpets digestive glands using the DNeasy PowerSoil Kit (Qiagen, Hilden, Germany), with minor adjustments to the manufacturer’s protocol: the homogenization step was performed with a FastPrep instrument (MP Biomedicals, Irvine, CA, USA) at 5.5 movements per sec for 1 min, and the elution step was preceded by a 5-min incubation at 4 °C [33].

All feces collected from each sample were used for DNA extraction. Seawater samples (1 L) were filtered onto 0.22 μm cellulose nitrate membrane filters (Sartorius, ø 47 mm) and stored at −20 °C until processing; the entire membrane filters were used for DNA extraction. The top 1 cm of each sterile corer used for sediments collection was carefully extruded and stored at −20 °C, and 1 g of this sediment was used for DNA extraction. DNA from sea bream feces and tissues, seawater, and sediment samples was extracted using the DNeasy PowerSoil Kit (Qiagen) as previously described [14,34]. Extracted DNA samples were quantified with NanoDrop ND-1000 (NanoDrop Technologies, Wilmington, DE, USA) and stored at −20 °C until further processing.

PCR amplification of the V3-V4 hypervariable region of the 16S rRNA gene was carried out in a 50-μL final volume containing 25 ng of microbial DNA, 2X KAPA HiFi HotStart ReadyMix (Roche, Basel, Switzerland), and 200 nmol/L of 341F and 785R primers carrying Illumina overhang adapter sequences. The thermal cycle consisted of 3 min at 95 °C, 30 cycles of 30 s at 95 °C, 30 s at 55 °C, and 30 s at 72 °C, and a final 5-min step at 72 °C [33,35]. PCR products were purified with Agencourt AMPure XP magnetic beads (Beckman Coulter, Brea, CA, USA). Indexed libraries were prepared by limited-cycle PCR with Nextera technology and cleaned-up as described above. Libraries were normalized to 4 nM and pooled. The sample pool was denatured with 0.2 N NaOH and diluted to a final concentration of 6 pM with a 20% PhiX control. Sequencing was performed on an Illumina MiSeq platform using a 2 × 250 bp paired-end protocol, according to the manufacturer’s instructions (Illumina, San Diego, CA, USA).

### 2.3. Bioinformatics and Statistics

A pipeline combining PANDAseq [36] and QIIME 2 [37] was used to process raw sequences. High-quality reads (min/max length = 350/550 bp) were retained using the “fastq filter” function of Usearch11 [38]. In particular, based on the phred Q score probabilities, reads with an expected error per base E = 0.03 (i.e., 3 expected errors every 100 bases) were discarded. Given its stringency, this algorithm is also very effective in singletons removal, due to their likely high error rate. Then, the reads resulting from the quality filtering were binned into amplicon sequence variants (ASVs) using DADA2 [39], as described by D’Amico et al. [40]. Taxonomy assignment was performed using the VSEARCH algorithm [41] and the SILVA database (December 2017 release) [42]. All the sequences assigned to eukaryotes (i.e., chloroplasts and mitochondria) or unassigned were discarded. Two different metrics were used to evaluate alpha diversity—Faith’s Phylogenetic Diversity (Faith_pd) [43] and the number of observed ASVs. Beta diversity was estimated by computing the unweighted UniFrac distance. To further characterize the compositional specificities of the limpets digestive gland microbiome at aquaculture and control sites in the context of their respective marine metacommunities, PANDAseq assembled paired-end reads were also processed with the QIIME1 [44] pipeline for OTUs (Operational Taxonomic Units) clustering based on a 97% similarity threshold, with taxonomy assignment performed using the SILVA database. As above, all the sequences assigned to eukaryotes or unassigned were discarded.

All statistical analyses were performed using the R software (R Core Team; https://www.R-project.org (last access: 10 January 2021)), v. 3.6.1, with the packages “Made4” [45] and “vegan” (https://cran.r-project.org/web/packages/vegan/index.html (last access: 10 January 2021)), except for environmental parameters that were compared between sites using Mann–Whitney, which was computed with IBM SPSS Statistics 26.0 (IBM Corporation, Armonk, NY, USA). Ternary plots were prepared using the R packages “ggtern” [46], “PMCMR” [47], and “vcd” [48]. Unweighted UniFrac distances were plotted using the vegan package, and the data separation in the Principal Coordinates Analysis (PCoA) was tested using a permutation test with pseudo-F ratios (function “adonis” in the vegan package). The Wilcoxon rank-sum test was used to assess differences in alpha diversity and ternary plots, and the Kruskal–Wallis test was used for testing OTUs separation. *p*-values were corrected for multiple testing with the Benjamini–Hochberg method, with a false discovery rate (FDR) ≤ 0.05 considered statistically significant.

## 3. Results

### 3.1. Diversity and Compositional Structure of the Marine Microbial Communities at Aquaculture and Control Site in the Licata Harbor

Sequencing of the V3-V4 hypervariable region of the 16S rRNA gene from the total microbial DNA resulted in 50 samples, from aquaculture and control sites, producing a high-quality output. These included three sediment and three seawater samples, 25 limpet digestive glands (DG), and 19 sea bream samples, consisting of 12 gut samples, three gill samples, and four skin samples. The number of high-quality reads in the samples ranged between 1690 and 20,252 per sample, with a subsequent normalization to the lowest number of reads (1690), resulting in 6450 ASVs and 8701 OTUs.

**Figure 2:**
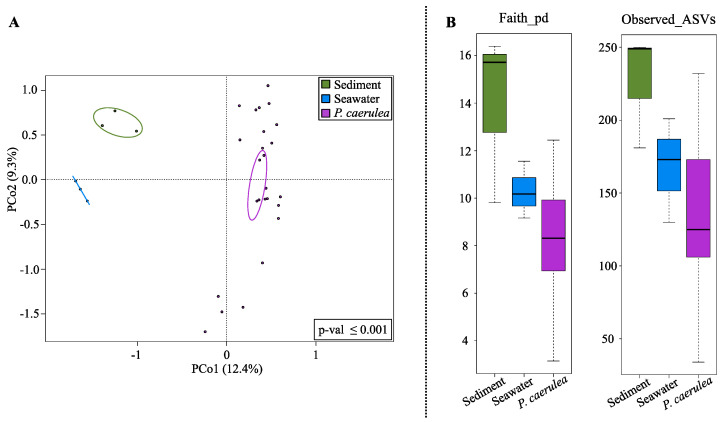
Alpha and beta diversity comparison between *P. caerulea* and the surrounding environment. (**A**) Principal Coordinates Analysis (PCoA) based on the unweighted UniFrac distances between microbial profiles of sediments, seawater, and limpets, showing a strong separation between the groups (permutation test with pseudo-F ratio, *p*-value ≤ 0.001). The percentage of variance in the dataset explained by each axis, first and second principal component (PCo1 and PCo2), is 12.4% and 9.3%, respectively. Ellipses include the 95% confidence area based on the standard error of the weighted average of sample coordinates. (**B**) Box-and-whiskers distribution of Faith’s Phylogenetic Diversity (Faith_pd) and number of observed amplicon sequence variants (ASVs) as metrics of alpha diversity.

According to our data, the overall structure of the limpet DG microbiome segregated from that of seawater and sediments microecosystems, as shown by the Principal Coordinates Analysis (PCoA) based on the unweighted UniFrac distances (Figure 2A) (permutation test with pseudo-F ratio, *p*-value ≤ 0.001). The *P. caerulea* DG microbiome was also characterized by a lower diversity with respect to environmental communities (seawater and sediments) (Figure 2B), although the trend did not always reach the statistical significance (Wilcoxon rank-sum test controlled for multiple testing using FDR; limpets vs. sediment *p*-value ≤ 0.05, limpets vs. seawater *p*-value 0.09 and 0.2 for Faith PD index and number of observed ASVs, respectively).

**Figure 3:**
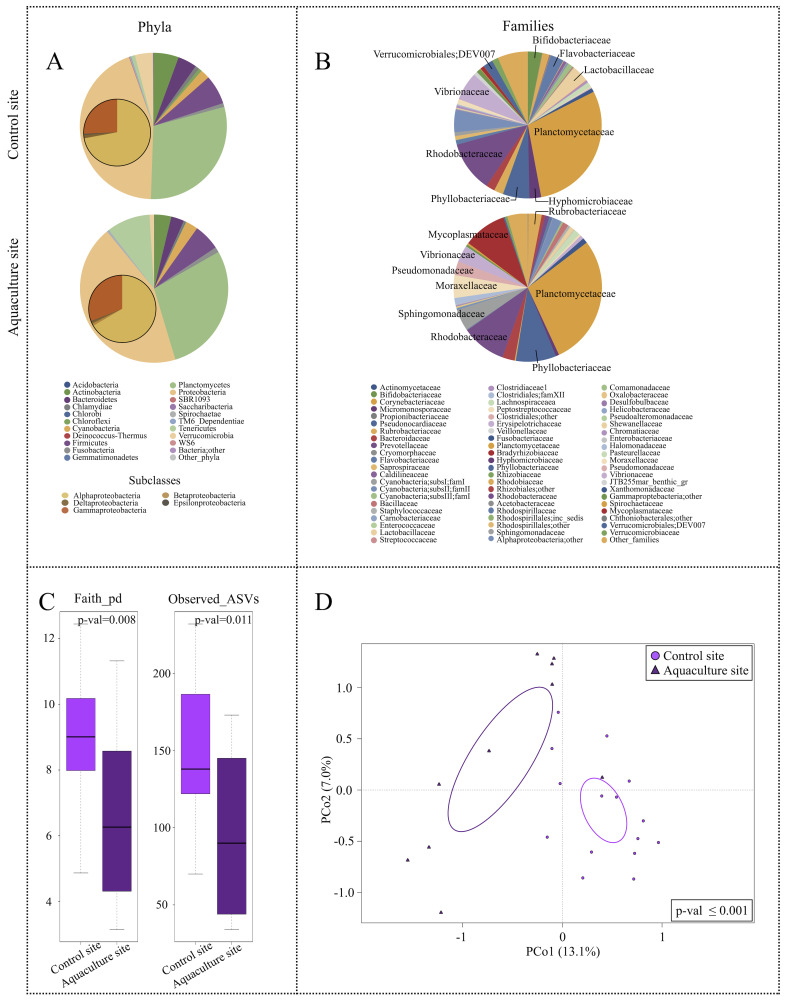
Overall description of *P. caerulea* microbial communities and alpha and beta diversity comparison between *P. caerulea* from control and aquaculture sites. Pie charts summarizing the phylum (**A**) and family (**B**) level microbiota composition of *P. caerulea* in the two sampling sites. Phyla with relative abundance >0.5% in at least one sample and families with relative abundance >2% in at least 10% of samples are represented. Proteobacteria classes are expanded on the respective pie chart phylum slice. (**C**) Box-and-whiskers distribution of the alpha diversity metrics Faith’s Phylogenetic Diversity (Faith_pd) and number of observed ASVs. (**D**) Principal Coordinates Analysis (PCoA) based on the unweighted UniFrac distances between microbial profiles of *P. caerulea* in the two sampling sites shows a strong separation between the groups (permutation test with pseudo-F ratio, *p*-value ≤ 0.001). The percentage of variance in the dataset explained by each axis, first and second principal component (PCo1 and PCo2), is 13.1% and 7.0%, respectively. Ellipses include 95% confidence area based on the standard error of the weighted average of sample coordinates.

Concerning their phylogenetic composition, the microbial assemblages associated with the three types of samples showed a characteristic layout in terms of dominant and subdominant components, with a specific declination according to the sampling site. Particularly, the microbiome of *P. caerulea* DG was characterized by two dominant phyla, Proteobacteria (mean relative abundance ± SD, 44.1 ± 12.6% and 43.7 ± 12.5% in limpets collected at the control and aquaculture sites, respectively) and Planctomycetes (29.7 ± 13.4% and 28.6 ± 22.4%). Alphaproteobacteria class represented the main fraction of Proteobacteria (72.0 ± 13.2% and 66.5 ± 23.0%). Relevant subdominant phyla (average relative abundance >1%) were Firmicutes (6.4 ± 11.6% and 5.7 ± 4.6%), Actinobacteria (5.5 ± 7.3% and 3.7 ± 7.1%), Bacteroidetes (4.2 ± 8.5% and 2.8 ± 3.2%), and Cyanobacteria (2.1 ± 1.4% and 2.7 ± 3.2%). In the control site, we also found Verrucomicrobia (3.9 ± 3.4%), whereas Tenericutes (9.5 ± 16.5%) and Fusobacteria (1.2 ± 2.7%) were only present in the aquaculture site (Figure 3A). The detailed compositional structure of water and sediments microbiomes at the aquaculture and control sites is available in Supplementary Figures S1 and S2. Briefly, both sediments and seawater were mainly characterized by Proteobacteria and Bacteroidetes at all sampling sites. In the aquaculture site, the two matrices also included members of the Firmicutes phylum, whereas Verrucomicrobia were only characteristic of seawater. Finally, the microbiomes associated with different tissues of farmed sea breams were also characterized (Figure S3). Our findings showed that all sea bream microbiomes were mainly characterized by Proteobacteria, with Firmicutes and Actinobacteria only represented in the gut samples.

### 3.2. Changes in the Limpet Microbiome and Surrounding Metacommunities at the Aquaculture Site in the Licata Harbor

In order to highlight the impact of aquaculture cages on the limpet DG microbiome, a PCoA of the unweighted UniFrac distances of the microbiome structures in individuals collected at aquaculture and control sites was performed (Figure 3D). Data indicated a significant segregation between the two ecosystems (permutation test with pseudo-F ratio, *p*-value ≤ 0.001), demonstrating that the phylogenetic composition of P. caerulea DG microbiome changed at the aquaculture site. Furthermore, DG samples collected in the aquaculture site showed a significantly lower alpha diversity, as shown by two different metrics (FDR-corrected Wilcoxon rank-sum test, *p*-value 0.008 and 0.01 for Faith PD index and number of observed ASVs, respectively) (Figure 3C). For what concerns the main DG microbiome compositional specificities, limpets at the aquaculture site were characterized by a higher relative abundance in members belonging to the family Mycoplasmataceae (9.4 ± 16.5% in samples from the cages vs. 0.8 ± 3.3% in controls). Conversely, among the subdominant components, the DG microbiome of individuals at the aquaculture site was significantly depleted in members of the uncultured Verrucomicrobiales group DEV007, showing a relative abundance (r.a.) of 0.3 ± 0.5% compared to 2.1 ± 1.9% observed in controls (FDR-corrected Wilcoxon rank-sum test, *p*-value = 0.007) (Figure 3B).

**Figure 4:**
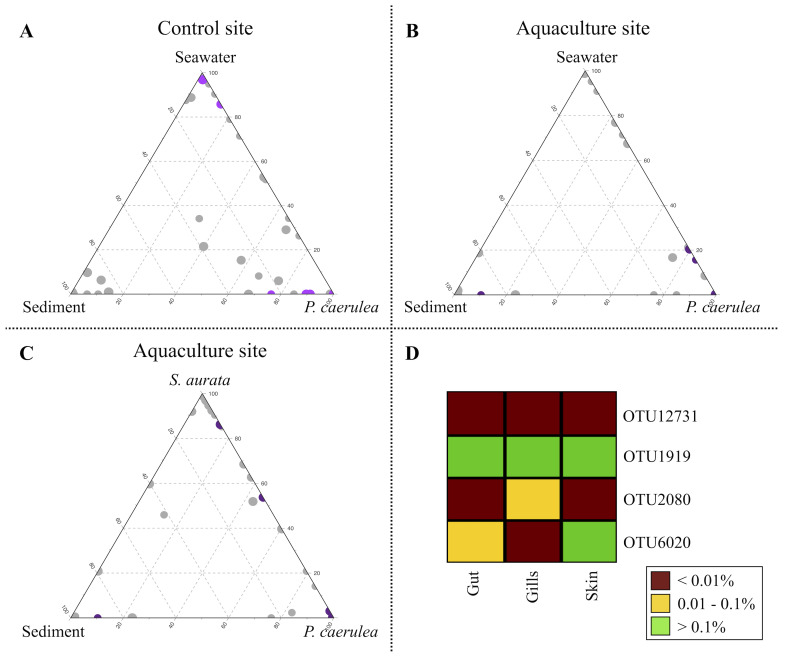
Impact of aquaculture cage proximity on *P. caerulea* microbiome at 97% similarity OTUs (Operational Taxonomic Units) level. (**A**–**C**) Ternary plots of all OTUs detected in the dataset with relative abundance >0.5% in at least one sample. Each circle represents one OTU, and the size is proportional to the weighted relative abundance. The position of each circle in the graphs represents its propensity toward the different ecosystems at the edges. (**A**) Purple circles represent the 22 OTUs whose mean relative abundance was significantly higher in the *P. caerulea* control site (false discovery rate (FDR)-corrected Wilcoxon rank-sum test, *p*-value ≤ 0.05). (**B**,**C**) Purple circles represent the 28 OTUs whose mean relative abundance was significantly higher in the *P. caerulea* aquaculture site (FDR-corrected Wilcoxon rank-sum test, *p*-value ≤ 0.05). (**D**) The heatmap represents the differential distribution of the OTUs shared between *P. caerulea* and farmed sea breams in the fish ecosystems (gut, gills, and skin).

A re-analysis of the sequences using a different strategy (UCLUST algorithm [38] with 97% similarity threshold) was subsequently carried out to characterize the compositional specificities of the limpet DG microbiome at the aquaculture and control site in the context of their respective marine metacommunities. The 97%-similarity threshold allowed us to obtain a cluster of sequences (OTUs), possibly ascribable to small groups of species, that could play specific ecological roles within and across the analyzed ecosystems. Among the obtained 97%-similarity OTUs, we filtered those detected with a relative abundance >0.5% in at least one microbiome sample type (limpets, seawater, sediments, and fish gut, gills, and skin). The resulting subset of 192 OTUs was used for the production of ternary plots to highlight the OTUs ecological propensity toward the different local microbial communities (Figure 4A–C). While for the control site, a single ternary plot was generated (Figure 4A), considering local water, sediments, and the DG from limpets, for the aquaculture site, two ternary plots were created, the first matching the one from the control site (Figure 4B) and the second in which seawater was substituted by *S. aurata* as the source ecosystem (Figure 4C). Furthermore, 50 OTUs showing a significantly different relative abundance in the DG microbiome from individuals collected at the aquaculture and control sites were identified (FDR-corrected Wilcoxon rank-sum test, *p*-value ≤ 0.05). Among these, 22 OTUs were more abundant in the control site (colored in purple in Figure 4A), whereas 28 OTUs showed a significant opposite trend (in purple in Figure 4B,C). The highest alignment scores against the NCBI 16S rRNA database of these OTUs are reported in Table S1.

Focusing on the 50 OTUs differentiating the limpet DG microbiome at aquaculture and control sites, the majority of those enriched in the latter were exclusive of limpets (plotted at the “*P. caerulea*” vertex in Figure 4A) (OTUs 4667, 11135, 4454, 12220, 1496, 4069, 4330, 14127, 5331, 14154, 3304, 11232, 11155, 14091, 11445, 3555, and 4234). These OTUs were assigned to species typically isolated from marine environment (e.g., species belonging to genera Fodinicurvata, Rubinisphaera, and Roseibacillus) [49,50,51,52] or from marine organisms, such as *Amorphus coralli*, firstly isolated from coral mucus [53]. Other OTUs characterizing the DG from the limpets collected at the control site, such as OTU5034 (genus Robiginitalea), OTU2911 (Actibacter), and OTU2120 (Photobacterium), were shared between limpets and sediments (plotted along the bottom plane of the ternary plot), whereas OTU2289 (Psychrobacter) was shared between limpets and water, with higher relative abundance in the latter (closer to the “Seawater” vertex in the ternary plot). Finally, OTU1355 (Prochlorococcus) was shared among all three ecosystems, with a higher proportion in seawater. Similar to what observed for the control site, the majority of OTUs characteristic of the limpets DG microbiome at the aquaculture site were exclusive of limpets (OTUs 4187, 11247, 11243, 11205, 6912, 5244, 4203, 2259, 4097, 2073, 12731, 11913, 4065, 4965, 2118, 2077, 2154, 11213, 1397, 11152, 4465, 4305, 6020, 3237, and 6006). Amongst these OTUs, four were assigned to the genus Mycoplasma, one to Vibrio and one to Acinetobacter, potential human pathogens found in marine organisms [54,55,56,57,58]. Within the remaining OTUs characterizing the DG microbiome from limpets living in aquaculture proximity, we only found one OTU shared between limpets and sediments (OTU14234, belonging to the genus Sulfurovum), which is mainly present in sediments. Two OTUs were shared between limpets and seawater in the aquaculture site (OTU1919 and OTU2080, assigned to Staphylococcus and Psychrobacter, respectively), with a higher relative abundance in limpet. Finally, limpets and farmed fishes shared four out of the 28 OTUs characteristic of the limpets DG microbiome at the aquaculture site, of which two more present in fish (OTU1919, Staphylococcus, and OTU6020, Pseudomonas) and two enriched in limpets (OTU12731, Sphingomonas, and OTU2080, Psychrobacter) (Figure 4B,C). Of these four OTUs, OTU1919 and OTU12731 were present at low relative abundance in all fish tissues, whereas OTU2080 specifically belonged to the ecosystem and OTU6020 was more abundant on the skin of fishes (Figure 4D).

## 4. Discussion

The monitoring and preservation of coastal marine ecosystems are pivotal for the maintenance of the ecological and economical services that these environments provide, such as habitat provision, nutrient cycling, protection of the coast itself, and food provision through fishery and farming [23,59]. Aquaculture provides a relevant contribution to the food economy of Mediterranean countries. However, similarly to most of the human food production activities, mariculture influences environmental conditions in the surrounding water column, as evidenced by the decline in seawater pH and subsequent shifts in carbonate–bicarbonate equilibria highlighted in the current study (Table S2), with direct and indirect impacts on marine biota [1]. Particularly, the health and functionality of the marine and coastal ecosystems are tightly linked to the resident environmental microbiomes, as well as to the ones associated with local holobionts. However, research focused on the impact of marine aquaculture on the coastal marine microbiomes is still in its infancy. While a considerable amount of work has been performed to assess the impact of aquaculture practices on the underlying seafloor microbial communities [6,12,13,14,15,16,17], very few preliminary data have been provided linking the presence of fish farming cages to variations in the microbiome of benthic organisms living in close proximity [24]. In this scenario, we explored the impact of gilthead sea bream cage farming in the Licata harbor, Sicily, Italy, on the microbiome of locally dwelling wild species, by using a commonly found grazer gastropod (the limpet *Patella caerulea*) as a representative organism. In particular, we explained the variations of the limpet DG microbiomes at the aquaculture and control sites in the context of parallel changes in the local marine metacommunities, including water, sediments, and farmed fish microbiomes.

In spite of being a crucial keystone species in coastal environments, very little information is available on the microbiome of limpets up to now, with the exception of a first exploration of the microbiome of *P. pellucida*, which is a prevalently Atlantic species that mostly parasitizes brown algae stems [60]. Consistently, the DG microbiome of *P. caerulea* analyzed in our work was dominated by Proteobacteria, but the most abundant class was Alphaproteobacteria instead of Betaproteobacteria, as reported for *P. pellucida*. Differences in proteobacterial classes could be related to several factors, including the different sustaining strategy of the two Patella species (with *P. caerulea* grazing on hard material algal coverage vs. *P. pellucida* parasitizing a single algal species), as well as the different environmental conditions characterizing the sampling areas (warm, shallow Mediterranean waters vs. Atlantic cold water). In addition, while *P. pellucida* harbored a large amount of Firmicutes, the second most abundant phylum in *P. caerulea* was Planctomycetes. The DG microbiome of *P. caerulea* was significantly different from the microbiomes in the surrounding environment (water and nearby seafloor samples), confirming previous studies on aquatic holobionts and their ability to operate a non-neutral selection process of microbes from the surrounding environment [22,61,62,63].

In our work, we were successful in demonstrating that limpets dwelling in proximity of aquaculture cages harbored a different DG microbiome compared to gastropods of the same species collected on distant rocky shores. Such difference was already evident when looking at the microbiome phylogenetic structure at the phylum level, with Tenericutes largely abundant in samples collected on the cages, whereas samples from the rocky shores were enriched in Verrucomicrobia. Frequently in cooperation with Planctomycetes [64], members of Verrucomicrobia are capable of processing decaying organic materials and polysaccharides [65,66]. Several studies have highlighted their symbiotic lifestyle in marine invertebrates with recent findings showing metabolic adaptations enabling a more efficient utilization of specific carbon sources present in the host [22,64,67]. However, we observed that the proximity to the aquaculture site was associated with the reduction of the Verrucomicrobia uncultured family DEV007 in the limpet microbiome of limpets. DEV007 is a marine group of bacteria recently pointed out as particularly sensitive to metal pollution in surface waters and marine sediments [68,69]. Its decrease in limpets growing in adhesion to the aquaculture cage might be related to the increase in heavy metal accumulation that often accompanies aquaculture practices [1], which can indirectly affect the most sensitive species in the microbiome of nearby wild organisms. Conversely, among the discriminant OTUs enriched in the microbiome of limpets collected on the aquaculture cage, we could find two OTUs putatively assigned to environmental bacteria that are instead reported as able to tolerate heavy metal pollution, namely *Acinetobacter guillouie* and *Mesorhizobium camelthorni* [70,71].

In addition to heavy metal contamination, the accumulation of organic matter on the seafloor beneath fish cages is considered one of the major impacts of aquaculture and may lead to a consequent depletion in oxygen availability in sediments, as well as to an increase in toxic products, such as sulfide and ammonium. Several studies have reported on the occurrence of sulfidobacteria in aquaculture, or nearby water, highlighting the potential importance of this taxa in the sulfur cycling within these systems [72]. Another bacterial species thriving in highly sulfidic fish-farm sediments is *Sulfurovum lithotrophicum*, a chemolithoautotroph ε-Proteobacteria able to use S^0^ or S_2_O_3_^2−^ as electron donors and O_2_ or NO^3−^ as electron acceptors. *S. lithotrophicum* bacteria have been isolated in sediments from the oxic–anoxic interface where sulfides meet oxygenated seawater [73]. The retrieval of higher relative abundances of OTU4065, assigned to Sulfitobacter, and OTU14234, belonging to the genus Sulfurovum, in *P. caerulea* individuals from the aquaculture site, as well as the observed sharing of the latter OTU with the aquaculture sediments, is in line with these previous findings, suggesting that the microbiome of locally dwelling holobionts might respond to environmental changes caused by the aquaculture practice [22], i.e., heavy metal and sulfide accumulation, through the selection of environmental microorganisms allowing adaptive responses. Moreover, it was also shown that Sulfitobacter species might also have an inhibitory activity toward *Vibrio anguillarum*, which is an important fish pathogen [74]. Thus, it is tempting to hypothesize that limpets might be pushed to select this particular bacterial group within their microbiome as a protective agent toward pathogens potentially enriched in the aquaculture site.

In relation to this, the possible pathogen flow from farmed to wild organisms in mariculture has been pointed out as an unavoidable problem of this particular aquaculture practice. Mollusks and other non-fish scavengers persist in the vicinity of sea cages for longer period than wild fishes, making them a target for pathogen transfer [1]. We found that Mycoplasmataceae, the most abundant family within the phylum Tenericutes, tended to be more abundant in the DG microbiome from the limpets dwelling on the cages. Particularly, an OTU assigned to the genus Mycoplasma was the most relevant discriminating OTU between *P. cearulea* DG specimens taken at the two sites. Considering that many Mycoplasma species are parasitic or pathogenic to humans and other animals [54,75], these findings, together with the detection of OTUs assigned to potential fish pathogens from the genera Vibrio and Acinetobacter [55,56,57,58] as the significantly most abundant in the DG microbiome from the limpets at the aquaculture cages, confirm that pathogen transfer between farmed fishes and wild limpets is possible. However, it must also be pointed out that Tenericutes, and particularly Mycoplasma, have been consistently observed as abundant, core members of several aquatic organisms’ microbiome, mainly including bivalves, where they exhibit commensalism [76,77,78,79,80,81]. A possible involvement in mutually beneficial interactions with the host—likely by assisting an efficient processing of complex organic compounds, abundant at aquaculture sites—is being progressively assumed [81,82,83] and corroborates the idea of a possible role of fouling organisms in reducing the environmental impact of aquaculture [84]. Since the OTUs assigned to the genera Mycoplasma, Vibrio, and Acinetobacter enriched in the DG microbiome from *P. caerulea* at the aquaculture site were not detectable in farmed fish samples and no disease was reported at the fish farming plant at the moment of sampling, we could hypothesize that relationships such as commensalism, rather than parasitism or pathogenicity, occurred between *P. caerulea* and OTUs belonging to these genera. However, their detection in DG microbiomes from aquaculture still poses questions on their possible spread in the surrounding environment, in the nearby wild organisms as well as to humans, to which they are pathogens.

We also found two OTUs (12731 and 11913) assigned to the Sphingomonas genus, shared between limpets and fish, which were significantly more abundant in limpets at the aquaculture site. Sphingomonas is a bacterial genus commonly found in fish skin [85] and the gut microbiome, and in farmed sea breams specifically, as reported by Floris et al. [86] and Estruch et al. [87]. In particular, Sphingomonas has been reported as part of the fish beneficial microbiota [88], and strains of this genus isolated from the gut microbiome of gilthead sea bream exhibited antibacterial activity against fish pathogens, such as *Vibrio alginolyticus* and *Photobacterium damselae* [89]. Thus, it is tempting to speculate that the observed enrichment in these microorganisms in the DG microbiome of *P. caerulea* growing in the aquaculture site represents a protective feature, resulting from an adaptive selection of protective microbiome components from the farmed fish.

The study presented here has a preliminary nature, and it cannot be excluded that our findings might specifically be related to the considered model (i.e., the selected fish species, the selected wild grazing organism, as well as the specific Licata harbor environment). However, it represents a successful application of an unprecedented approach by considering a locally dwelling holobiont, i.e., the limpet, as a “proxy” for the pervasive effects that aquaculture cages can exert on wildlife. Indeed, even if confirmation on different temporal and spatial scales is still needed, our results support the hypothesis that aquaculture impacts the surrounding microbial communities, not only the ones from underlying sediments but also the microbiome of locally dwelling wild holobionts. According to our data, this seems to happen either directly through the transfer of microorganisms from the farmed fish microbiomes to the microbiomes of local wild holobionts or indirectly with the aquaculture practice changing the chemical conditions of the environment, resulting in the selection of specific microbiome components in the local marine metacommunities. Changes in the *P. caerulea* DG microbiome in individuals growing at the aquaculture site involve the loss of several microorganisms assigned to bacteria commonly found in wild marine organisms, as well as the concomitant acquisition of potential fish and human pathogens and parasites, resulting in an overall significantly lower ecosystem biodiversity. Even if these features generally mirror dysbiotic changes, we were also able to observe possible adaptive microbiome variations, showing the inherent potential of holobiont microbiomes in enabling host adaptation to the changing environment, including changes resulting from marine aquaculture practices. Our study demonstrates the impact of using wider metacommunity approaches through the use of sedentary organisms as a proxy for metacommunity changes. Future studies may assess the viability and efficacy of other sedentary organisms that dwell on aquaculture cages (e.g., bivalves, tunicates polychaetes, and fouling algae) to assess changes to different components of an ecosystem created by human activity, possibly using a longitudinal approach along the aquaculture productive cycles.

## Data Availability

Sequence reads were deposited in the National Center for Biotechnology Information Sequence Read Archive (NCBI SRA; BioProject ID PRJNA692072).

## Supplementary Materials

The following are available online at https://www.mdpi.com/2076-2607/9/2/455/s1. Figure S1: Pie charts summarizing the phylum (A) and family (B) level microbiota composition of sediment samples in the two sampling sites. Phyla with relative abundance >0.5% in at least one sample and families with relative abundance >2% in at least 10% of samples are represented. Proteobacteria classes are expanded on the respective pie chart phylum slice, Figure S2: Pie charts summarizing the phylum (A) and family (B) level microbiota composition of seawater samples in the two sampling sites. Phyla with relative abundance >0.5% in at least one sample and families with relative abundance >2% in at least 10% of samples are represented. Proteobacteria classes are expanded on the respective pie chart phylum slice, Figure S3: Pie charts summarizing the phylum (A) and family (B) level microbiota composition of *S. aurata* samples in the three fish districts (gut, gills, and skin). Phyla with relative abundance >0.5% in at least one sample and families with relative abundance >2% in at least 10% of samples are represented. Proteobacteria classes are expanded on the respective pie chart phylum slice, Table S1: Ecological distribution and highest alignment score against NCBI 16S rRNA database of OTUs showing a significantly higher mean relative abundance in the limpets collected from control site with respect to those from the aquaculture cage and vice versa. *p*-values were calculated for the two *P. caerulea* groups (control vs. aquaculture, FDR-corrected Wilcoxon rank-sum test, *p*-value ≤ 0.05). Species, genera, or families are retrieved on the BLAST column based on the last common taxonomic level shared between all BLAST best hits, Table S2: Seawater environmental data. Measurements (*n* = 6 per site) are shown for the control and aquaculture sites. Measured parameters, namely T, pH, TA, and salinity (38‰ in control and 34‰ in the in the aquaculture site) were used to calculate the carbonate chemistry parameters through CO2SYS Software. T = Temperature; TA = Total Alkalinity; pCO2 = carbon dioxide partial pressure; HCO_3_^−^ = bicarbonate; CO_3_^2−^ = carbonate; DIC = dissolved inorganic carbon; Ωarag = aragonite saturation; NS = not significant; ** *p* < 0.01, Mann–Whitney test. In brackets the 95% confidence interval.
