# Supplementary material for: Impact of Marine Aquaculture on the Microbiome Associated with Nearby Holobionts: The Case of Patella caerulea Living in Proximity of Sea Bream Aquaculture Cages
Source: Microorganisms. 2021 Feb 22;9(2):455. doi: 10.3390/microorganisms9020455 (PMC7927081; doi:10.3390/microorganisms9020455)
Supplement: Supplementary file 1 [file microorganisms-09-00455-s001.pdf]

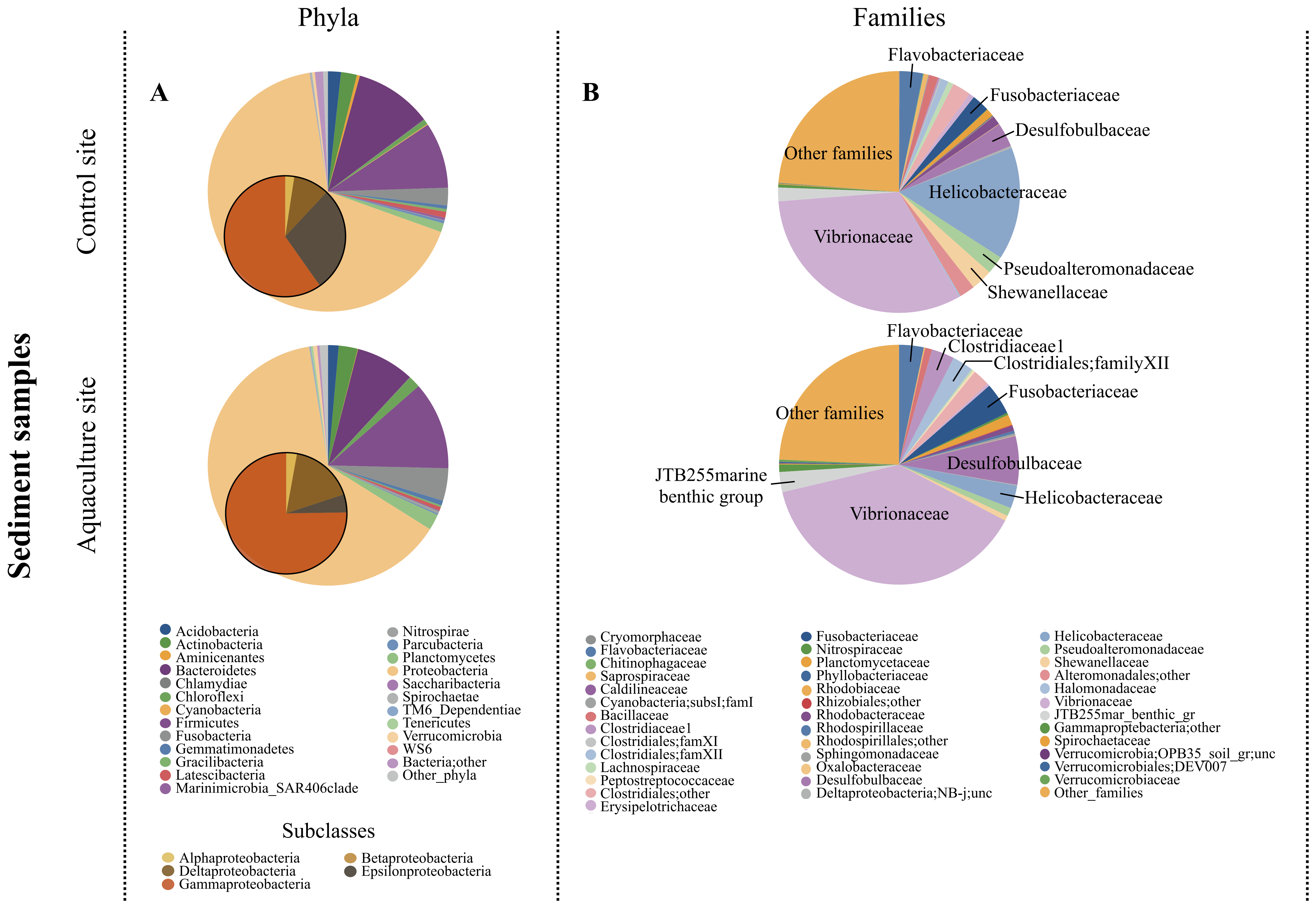

Supplementary figure S1 - Pie charts summarizing the phylum (A) and family (B) level microbiota composition of sediment samples in the two sampling sites. Phyla with relative abundance > 0.5% in at least one sample and families with relative abundance > 2% in at least 10% of samples are represented. Proteobacteria classes are expanded on the respective pie chart phylum slice. subs=subset; fam=family; unc=uncultured; inc=incertae; mar=marine; gr=group.

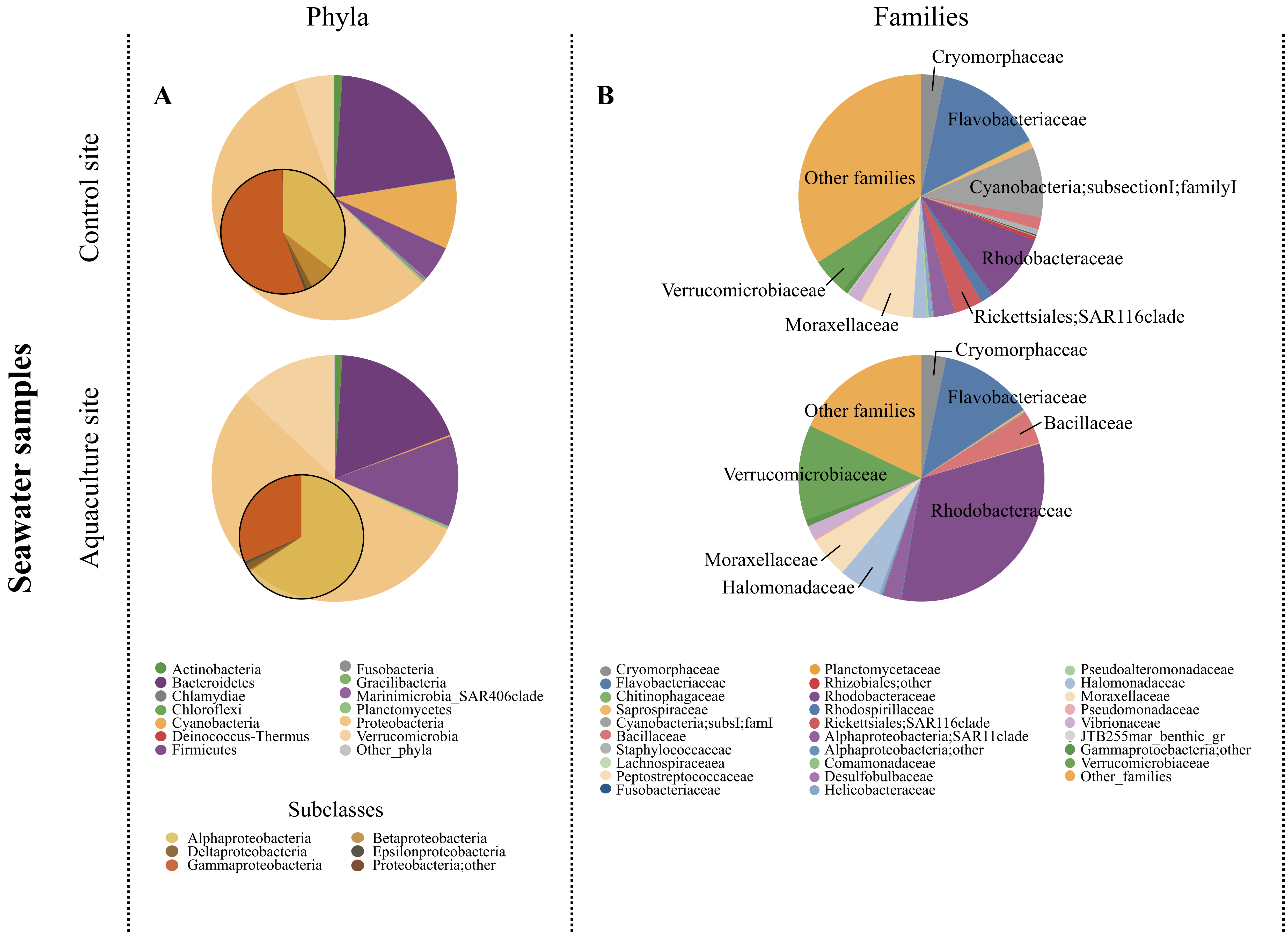

Supplementary figure S2 - Pie charts summarizing the phylum (A) and family (B) level microbiota composition of seawater samples in the two sampling sites. Phyla with relative abundance > 0.5% in at least one sample and families with relative abundance > 2% in at least 10% of samples are represented. Proteobacteria classes are expanded on the respective pie chart phylum slice. subs=subset; fam=family; unc=uncultured; inc=incertae; mar=marine; gr=group.

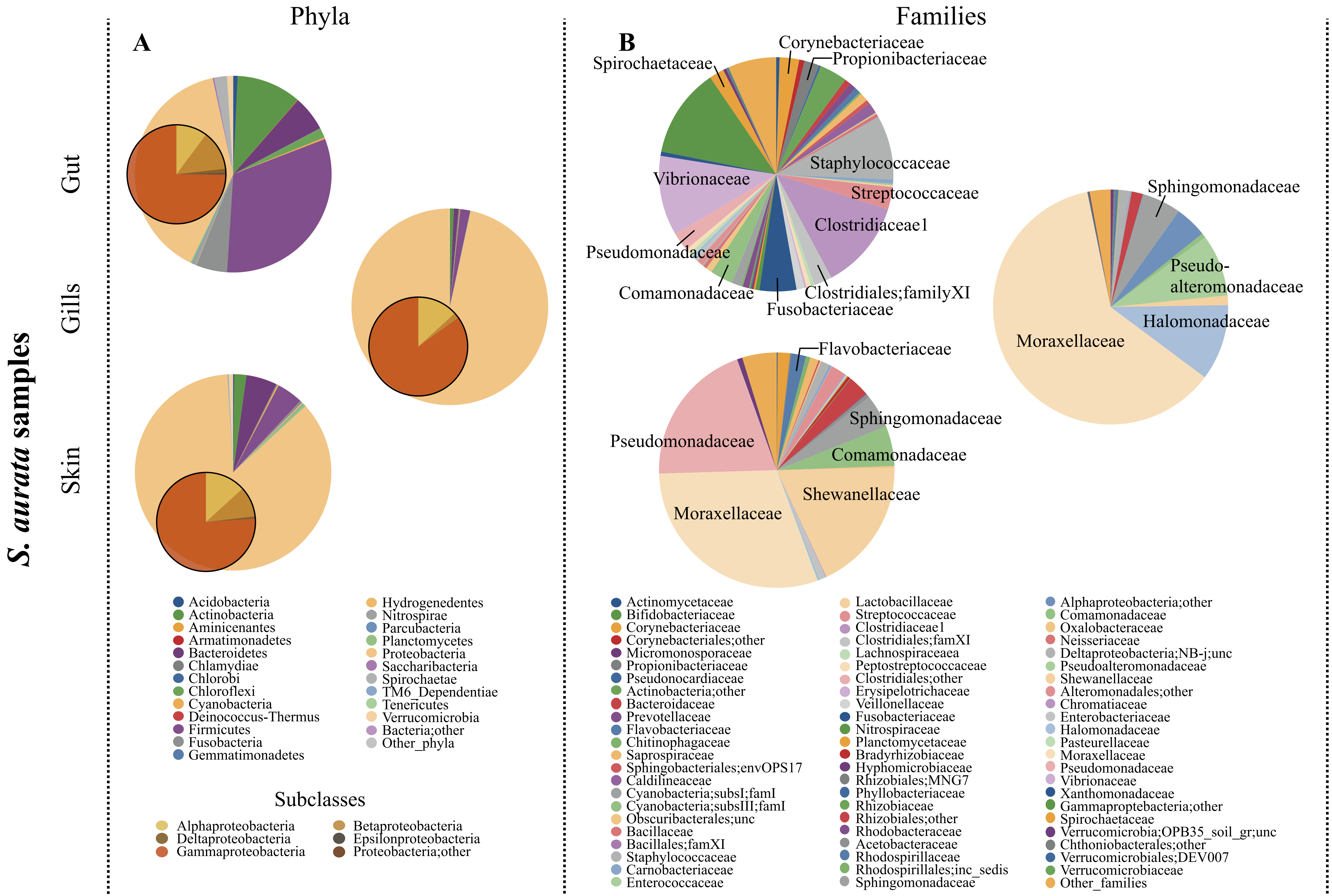

Supplementary figure S3 - Pie charts summarizing the phylum (A) and family (B) level microbiota composition of *S. aurata* samples in the three fish districts (feces, gills and skin). Phyla with relative abundance > 0.5% in at least one sample and families with relative abundance > 2% in at least 10% of samples are represented. Proteobacteria classes are expanded on the respective pie chart phylum slice. subs=subset; fam=family; unc=uncultured; inc=incertae; mar=marine; gr=group.

	OTU_ID	BLAST	Control site r.a. (%)			Aquaculture site r.a. (%)				p-value
			P. caerulea	Sediment	Seawater	*P. caerulea*	Sediment	Seawater	Fish	
Control site	4667	*Fodinicurvata halophila*	0.19	0.00	0.00	0.00	0.00	0.00	0.00	0.004
	11135	*Lactobacillus johnsonii*	0.10	0.00	0.00	0.04	0.00	0.00	0.00	0.05
	4454	*Fluviibacterium aquatile*	0.22	0.00	0.00	0.00	0.00	0.00	0.00	0.0004
	5034	*Robiginitalea biformata*	0.06	0.02	0.00	0.00	0.00	0.00	0.00	0.03
	12220	*Rhodobacteraceae*	0.13	0.00	0.00	0.00	0.00	0.00	0.00	0.0001
	1496	*Agaricicola taiwanensis*	0.48	0.00	0.00	0.02	0.00	0.00	0.00	0.01
	4069	*Amorphus coralli*	0.10	0.00	0.00	0.01	0.00	0.00	0.00	0.02
	4330	*Ochrobactrum oryzae*	0.11	0.00	0.00	0.00	0.00	0.00	0.00	0.02
	14127	*Rubinisphaera brasiliensis*	0.09	0.00	0.00	0.00	0.00	0.00	0.00	0.02
	5331	*Roseibacillus ponti*	0.06	0.00	0.00	0.00	0.00	0.00	0.00	0.0008
	2911	*Actibacter sediminis*	0.29	0.03	0.00	0.00	0.13	0.00	0.00	0.0004
	2289	*Psychrobacter celer*	0.03	0.00	0.17	0.00	0.00	0.07	0.06	0.008
	14154	*Phyllobacterium*	0.20	0.00	0.00	0.06	0.00	0.00	0.00	0.04
	3304	*Stappia taiwanensis*	0.31	0.00	0.00	0.00	0.00	0.00	0.00	0.00002
	11232	*Rubinisphaera italica*	0.07	0.00	0.00	0.00	0.00	0.00	0.00	0.03
	1355	*Prochlorococcus marinus*	0.02	0.02	1.18	0.00	0.01	1.88	0.01	0.007
	11155	*Lactobacillus rhamnosus*	0.24	0.00	0.00	0.00	0.00	0.00	0.00	0.006
	14091	*Bifidobacterium bifidum*	0.83	0.00	0.00	0.02	0.00	0.00	0.00	0.003
	11445	*Ahrensia kielensis*	0.13	0.00	0.00	0.00	0.00	0.00	0.00	0.0007
	3555	*Bifidobacterium longum*	0.50	0.00	0.00	0.01	0.00	0.00	0.00	0.0004
	2120	*Photobacterium swingsii*	0.23	0.02	0.00	0.01	0.00	0.00	0.00	0.003
	4234	*Hyphomicrobium*	0.07	0.00	0.00	0.00	0.00	0.00	0.00	0.03
Aquaculture site	4187	*Phyllobacteriaceae*	0.00	0.00	0.00	0.13	0.00	0.00	0.00	0.0003
	11247	*Rhodopirellula*	0.00	0.00	0.00	0.13	0.00	0.00	0.00	0.03
	11243	*Alienimonas californiensis*	0.00	0.00	0.00	0.35	0.00	0.00	0.00	0.001
	11205	*Wenxinia marina*	0.00	0.00	0.00	0.09	0.00	0.00	0.00	0.003
	6912	*Mycoplasma mobile*	0.45	0.00	0.00	2.92	0.00	0.00	0.00	0.0004
	5244	*Acinetobacter guillouiae*	0.03	0.00	0.00	0.16	0.00	0.00	0.00	0.04
	4203	*Mesorhizobium thiogangeticum*	0.00	0.00	0.00	0.36	0.00	0.00	0.00	0.003
	2259	*Mycoplasma mobile*	0.02	0.00	0.00	0.19	0.00	0.00	0.00	0.002
	4097	*Mesorhizobium camelthorni*	0.00	0.00	0.00	0.37	0.00	0.00	0.00	0.0002
	2073	*Halomonas*	0.05	0.00	0.00	0.18	0.00	0.00	0.00	0.05
	12731	*Sphingomonas*	0.37	0.00	0.00	1.96	0.00	0.00	0.00	0.001
	11913	*Sphingomonas*	0.06	0.00	0.00	0.40	0.00	0.00	0.00	0.0006
	4065	*Sulfitobacter pontiacus*	0.00	0.00	0.03	0.20	0.00	0.00	0.00	0.03
	4965	*Rhodopirellula baltica*	0.00	0.00	0.00	0.07	0.00	0.00	0.00	0.03
	2118	*Vibrio atypicus*	0.42	0.01	0.00	0.63	0.00	0.00	0.00	0.02
	1919	*Staphylococcus*	0.01	0.01	0.01	0.06	0.00	0.01	0.39	0.04
	2077	*Mycoplasma mobile*	0.05	0.00	0.00	0.33	0.00	0.00	0.00	0.005
	2154	*Mycoplasma mobile*	0.03	0.00	0.00	0.16	0.00	0.00	0.00	0.002
	11213	*Rubinisphaera italica*	0.01	0.00	0.00	0.27	0.00	0.00	0.00	0.005
	1397	*Foliisarcina bertiogensis*	0.00	0.00	0.00	0.10	0.00	0.00	0.00	0.002
	11152	*Bacteroides*	0.00	0.00	0.00	0.08	0.00	0.00	0.00	0.006
	2080	*Psychrobacter marincola*	0.00	0.00	0.19	0.13	0.00	0.03	0.00	0.02
	14234	*Sulfurovum lithotrophicum*	0.00	0.82	0.00	0.01	0.08	0.00	0.00	0.01
	4465	*Phyllobacteriaceae*	0.00	0.00	0.00	0.27	0.00	0.00	0.00	0.0001
	4305	*Marimonas arenosa*	0.00	0.00	0.00	0.14	0.00	0.00	0.00	0.04
	6020	*Pseudomonas*	0.00	0.00	0.00	0.07	0.00	0.00	0.08	0.01
	3237	*Bythopirellula goksoyri*	0.00	0.00	0.00	0.13	0.00	0.00	0.00	0.03
	6006	*Gimesia maris*	0.02	0.00	0.00	0.14	0.00	0.00	0.00	0.05

Supplementary table S1 - Ecological distribution and highest score alignment against NCBI 16S rRNA database of OTUs showing a significantly higher mean relative abundance in the limpets collected from control site with respect to those from the aquaculture cage and vice versa. P-values were calculated for the two *P. caerulea* groups (control vs. aquaculture, FDR-corrected Wilcoxon rank-sum test, p-value ≤ 0.05). Species, genera or families are retrieved on the BLAST column based on the last common taxonomic level shared between all BLAST best hits.

Supplementary table S2 - Seawater environmental data. Measurements (N = 6 per site) are shown for the control and aquaculture sites. Measured parameters, namely T, pH, TA and salinity (38‰ in control and 34‰ in the in the aquaculture site) were used to calculate the carbonate chemistry parameters through CO2SYS Software. T = Temperature; TA = Total Alkalinity; pCO2 = carbon dioxide partial pressure; HCO_3^- = bicarbonate; CO_3^{2-} = carbonate; DIC = dissolved inorganic carbon; Ωarag = aragonite saturation; NS = not significant; **p<0.01, Mann-Whitney test. In brackets the 95% confidence interval.

	T (°C)	pH_{NBS}	TA (µmol kg^{-1})	pCO_2 (µatm)	HCO_3^- (µmol kg^{-1})	CO_3^{2-} (µmol kg^{-1})	DIC (µmol kg^{-1})	Ω_{arag}
Control	27.3	8.02	2319	667	1898	170	2086	2.67
	(26.9-27.6)	(8.00-8.04)	(2315-2324)	(620-714)	(1882-1914)	(164-177)	(2075-2097)	(2.57-2.76)
Aquaculture	24.8	7.96	2203	700	1877	132.1	2030	2.11
	(24.7-24.9)	(7.95-7.97)	(2191-2213)	(682-717)	(1872-1883)	(130-134)	(2026-2033)	(2.07-2.14)
Mann-Whitney	**	**	**	NS	**	**	**	**
